# Strategies to recruit people with selected nationalities for the interview survey GEDA Fokus

**DOI:** 10.1093/eurpub/ckac130.200

**Published:** 2022-10-25

**Authors:** C Koschollek, J Geerlings, M Bug, M Blume, K Kajikhina, C Hövener

**Affiliations:** 1 Department Epidemiology and Health Monitoring, Robert Koch Institute, Berlin, Germany; 2 Department Infectious Disease Epidemiology, Robert Koch Institute, Berlin, Germany

## Abstract

**Background:**

People with migration history (PMH) are underrepresented within health monitoring at Robert Koch Institute (RKI). To better describe the health status of PHM, the RKI is currently conducting the health interview survey GEDA Fokus with different migrant groups. Aim of this contribution is to present which sub-groups in this sequential mixed-mode survey design are reached by which mode.

**Methods:**

People aged 18-70 years with Croatian (hr), Italian (it), Polish (pl), Syrian (sy) or Turkish (tr) nationality were drawn out of 99 residents’ registration offices all over Germany (N = 33,436). Study persons were invited sequentially to participate online (saq-web), paper-based(saq-paper) and in person (CAPI) or by telephone (CATI) in Arabic, Croatian, German, Italian, Polish or Turkish. Saq-web was available in German only or bilingual. Data collection took place from November 2021 to April 2022. Per nationality, 1,200 participants were recruited.

**Results:**

As of 25th of April, 6,197 people took part, most often per saq-web (49%), which was less often used among participants aged ≥ 50 years (35% vs. 56%). Pl participants used saq-web most often in German only (55%), sy participants most often used the bilingual version (77%). Saq-paper was more often used by participants aged ≥ 50 years (41% vs. 22%) and less often by tr (21%) and sy (24%) participants. Participants with it nationality most often took part on their own initiative (saq) (92%), while tr (33%) and pl (28%) participants were recruited via personal contact (CAPI/ CATI) more often.

**Conclusions:**

Preliminary results show that offering multiple modes of administration helps to reach different sub-groups. Personal contact contributes to reach those not directly taking part on their own initiative. The opportunity to utilize the bilingual version of the questionnaire was well accepted, especially among those with presumably shorter duration of residence in Germany.

**Key messages:**

- Offering different modes of survey administration, including personal contact increases survey participation.
- In order to reach people with migration history in their heterogeneity the utilization of multilingual questionnaires is crucial.

